# From threat to challenge—Improving medical students’ stress response and communication skills performance through the combination of stress arousal reappraisal and preparatory worked example-based learning when breaking bad news to simulated patients: study protocol for a randomized controlled trial

**DOI:** 10.1186/s40359-023-01167-6

**Published:** 2023-05-10

**Authors:** Michel Bosshard, Felix Michael Schmitz, Sissel Guttormsen, Urs Markus Nater, Patrick Gomez, Christoph Berendonk

**Affiliations:** 1 Institute for Medical Education, University of Bern, Bern, Switzerland; 2 Department of Clinical and Health Psychology, University of Vienna, Vienna, Austria; 3 University Research Platform “Stress of life (SOLE) – Processes and Mechanisms underlying Everyday Life Stress”, University of Vienna, Vienna, Austria; 4 Center for Primary Care and Public Health (Unisanté), Department of Occupational and Environmental Health, University of Lausanne, Lausanne, Switzerland

**Keywords:** Breaking bad news, Stress, Arousal, Reappraisal, Worked example, Medical education, Psychophysiology, Challenge, Threat

## Abstract

**Background:**

Breaking bad news (BBN; e.g., delivering a cancer diagnosis) is perceived as one of the most demanding communication tasks in the medical field and associated with high levels of stress. Physicians’ increased stress in BBN encounters can negatively impact their communication performance, and in the long term, patient-related health outcomes. Although a growing body of literature acknowledges the stressful nature of BBN, little has been done to address this issue. Therefore, there is a need for appropriate tools to help physicians cope with their stress response, so that they can perform BBN at their best. In the present study, we implement the biopsychosocial model of challenge and threat as theoretical framework. According to this model, the balance between perceived situational demands and perceived coping resources determines whether a stressful performance situation, such as BBN, is experienced as challenge (resources > demands) or threat (resources < demands). Using two interventions, we aim to support medical students in shifting towards challenge-oriented stress responses and improved communication performance: (1) stress arousal reappraisal (SAR), which guides individuals to reinterpret their stress arousal as an adaptive and beneficial response for task performance; (2) worked examples (WE), which demonstrate how to BBN in a step-by-step manner, offering structure and promoting skill acquisition.

**Methods:**

In a randomized controlled trial with a 2 (SAR vs. control) x 2 (WE vs. control) between-subjects design, we will determine the effects of both interventions on stress response and BBN skills performance in *N* = 200 third-year medical students during a simulated BBN encounter. To identify students’ stress responses, we will assess their perceived coping resources and task demands, record their cardiovascular activity, and measure salivary parameters before, during, and after BBN encounters. Three trained raters will independently score students’ BBN skills performances.

**Discussion:**

Findings will provide unique insights into the psychophysiology of medical students who are tasked with BBN. Parameters can be understood more comprehensively from the challenge and threat perspective and linked to performance outcomes. If proven effective, the evaluated interventions could be incorporated into the curriculum of medical students and facilitate BBN skills acquisition.

**Trial registration:**

ClinicalTrials.gov (NCT05037318), September 8, 2021.

**Supplementary Information:**

The online version contains supplementary material available at 10.1186/s40359-023-01167-6.

## Background

### Breaking Bad News – an important and stressful communication task

Breaking bad news (BBN) in the medical field refers to the delivery of a serious diagnosis (e.g., cancer diagnosis, stillbirth) from a physician to a patient which is associated with sudden lifechanging consequences for the patient [1, 2]. Not every diagnosis is of the same severity, and how bad news is received by the patient depends on many individual characteristics (e.g., expectations, personality traits, social support) [3]. Therefore, patients’ emotional reactions to bad news can differ greatly—from perplexity, silence, and disbelief to intense displays of anger or sadness—and are thus very difficult for the physician to predict [1, 4]. Even physicians who are frequently confronted with BBN regard it as highly demanding and stressful, and most often feel overwhelmed, uncomfortable, or insufficiently prepared [5–9]. Increased psychophysiological stress levels are observed in physicians during BBN encounters [6, 8]. Importantly, these increased stress levels can already be seen in medical students learning how to BBN in role-play scenarios with simulated patients (SPs) [10, 11]. Increased stress, in return, can impair educational and clinical skills performances (e.g., communication performance) of medical students in particular [12]. When being confronted with a BBN task, stressed physicians might give false hope or even avoid disclosing the bad news [3, 13]. However, it is of utter importance that bad news is delivered appropriately—with empathy while not trivializing unpleasant facts [1]. It has been shown that the way bad news is communicated can affect patient-related outcomes in the long term (e.g., treatment adherence [14–16], recovery [17–19], psychological wellbeing [18, 20]). While a growing body of literature acknowledges the highly stressful nature of BBN and investigates best-practice in BBN (see [8] for a review), further exploration of medical students’ stress responses is needed and tools to help them manage their stress in this context have been widely neglected. Although time-intensive stress management programs have helped students cope with their stress in medical training [21], integrating such programs into the already overloaded curriculum of medical students is problematic. Moreover, such extensive stress interventions can lack acceptance due to the fact that the state of ‘being stressed’ is increasingly seen as normal among medical students and that suffering from it may be viewed as a form of weakness [22]. Hence, there is a need for low-threshold approaches that allow students both to identify stress and to deal with it appropriately without being stigmatized [23].

In the current study, we aim to make a significant contribution to the advancement of research by adopting the biopsychosocial model (BPSM) of challenge and threat as organizing framework to investigate the effects of stress arousal reappraisal and worked example-based BBN learning on the psychophysiological responses and communication skills performance of medical students tasked with BBN to SPs. We first introduce the BPSM of challenge and threat, then the stress arousal reappraisal intervention, and finally, the worked example-based BBN learning.

### The biopsychosocial model of challenge and threat

To understand medical students’ stress responses more holistically, we adopt the BPSM of challenge and threat as theoretical framework [24, 25]. The model builds on the premise that psychological stress states are embodied in measurable activity patterns of the cardiovascular system [26, 27]. The model is further specified to motivated performance situations, which require active (cognitive or behavioral) responses to achieve personally relevant outcomes (e.g., academic examinations, job interviews, public speaking) [24, 28, 29]. The active response and self-relevance lead to task engagement (indexed by an increased heart rate and a reduced pre-ejection period), which is a precondition for, and common across, the challenge-threat continuum [30]. Given task engagement, on the one hand, challenge emerges when individuals perceive their own resources (e.g., knowledge, abilities, familiarity) as higher than the situational demands (e.g., danger, effort, uncertainty). On the other hand, threat occurs when situational demands exceed perceived resources. The evaluation of resources and demands is an automatic and dynamic process (i.e., the evaluation can shift as the situation unfolds) [24, 25, 31, 32]. The resources-demands differential (i.e., perceived coping resources minus perceived task demands) is represented on a bipolar continuum, with challenge and threat as anchors, rather than distinct states. Therefore, relative differences in demands and resources can be interpreted as greater or lesser challenge and threat [33]. A more positive score reflects the task being evaluated as more of a challenge and less of a threat.

An important aspect of the BPSM of challenge and threat is that distinctive cardiovascular patterns manifest in challenge and threat states. Challenge states are accompanied by a lower total peripheral resistance (TPR; an index of net constriction vs. dilation in the vascular system) and increased cardiac output (CO; liters of blood pumped per minute) compared to threat states. In other words, not only does the heart pump more blood but the veins and arteries also expand to improve blood flow, resulting in a more efficient cardiovascular activity. This adaptation does not occur in threat states, meaning that the faster and stronger heart contractions during stress in fact increase TPR while not necessarily affecting CO [30, 34]. Together, the sum of CO and reverse scored TPR define the cardiovascular challenge-threat index. A higher cardiovascular challenge-threat index (higher CO, lower TPR) indicates a more adaptive challenge-oriented stress response. An advantage of the cardiovascular indices of challenge and threat is that they do not depend on an individual’s willingness or ability to accurately report on their appraisals [35, 36].

Recently, there has been increasing interest in understanding how other important physiological systems may vary along the challenge-threat continuum [28, 37]. It has been theorized that both challenge and threat states activate the sympathetic-adrenal-medullary (SAM) axis, whereas the hypothalamic-pituitary-adrenal (HPA) axis might be more responsive to threat states, in anticipation of failure and damage [30, 38–40].

Activation of the SAM axis increases the synthesis and release of catecholamines from the adrenal medulla—particularly epinephrine and norepinephrine—which in return trigger a multitude of cardiovascular adaptations. The main functions are to increase heart rate, reduce pre-ejection period, and constrict veins (to facilitate the return of oxygen-poor blood to the heart) [29]. Epinephrine further mediates adrenergic vasodilation (widening of blood vessels by relaxation of smooth muscles) [41]. These cardiovascular adaptations match the cardiovascular challenge pattern, which is suggested to be governed by the SAM activity. However, a downside of epinephrine and norepinephrine as indices of SAM activity is that they can only be measured reliably by blood taking. In this context, salivary alpha-amylase (sAA)—an enzyme secreted from the acinar cells of the salivary glands—has gained importance as a non-invasive marker for the activity of the locus coeruleus-norepinephrine system and peripheral SAM axis activity [42–46]. Previous research suggests that sAA may well indicate challenge and threat, whereby increased levels of sAA manifest in challenge states, although the authors acknowledge that this has yet to be confirmed by further research [47, 48]. The SAM axis is a fast-acting system and cardiovascular adaptations to stressors occur almost immediately.

In contrast, the main product of the HPA axis is the catabolic hormone cortisol, released from the adrenal cortex. It is theorized that cortisol dampens epinephrine-induced vasodilation, thereby increasing blood pressure when both axes are activated simultaneously [24, 39, 40]. In line with this, individuals in a cardiovascular threat state exhibited higher cortisol reactivity compared to individuals in a cardiovascular challenge state [49]. Activation of the HPA axis during acute stress also triggers the release of the anabolic hormone dehydroepiandrosterone (DHEA), which may play a protective factor during stress reaction and contribute to more favorable behavioral and emotional responses [50–56]. Elevated levels of DHEA compared to cortisol are further associated with resilience and thriving in stressful situations [57–59]. Therefore, the anabolic balance (i.e., the ratio of DHEA to cortisol) between the two hormones is of special interest, as they regulate each other and together could provide a sensitive indicator of challenge and threat [60]. While the HPA axis also responds instantly, observable adaptations and cortisol release take more time to manifest and to return to homeostasis.

To summarize, according to the BPSM of challenge and threat, an initial evaluation of personal resources and situational demands orchestrates downstream stress responses in the context of motivated performance situations (see Table 1). Crucially, previous research suggests that the resources-demands differential is amenable to interventions (e.g., [47, 61–63]) and, as shown in a recent meta-analysis [64], more adaptive challenge states are intertwined with increased task performance. In the present project, we propose stress arousal reappraisal and worked example-based learning interventions, that could be used to achieve more adaptive stress responses and communication performances in the BBN context.

**Table 1:** Psychophysiological adaptations depending on challenge and threat

Students in challenge state		Students in threat state
	**Psychological adaptations**	
Perceived coping resources higher than perceived task demands	Resources-demands differential	Perceived coping resources lower than perceived task demands
	**Neuroendocrine adaptations**	
Higher	Anabolic balance (DHEA/Cortisol)	Lower
Higher	sAA	Lower
	**Cardiovascular adaptations**	
Lower	TPR	Higher
Higher	CO	Lower
Better BBN communication	**Performance**	Worse BBN communication

*Note.* DHEA = dehydroepiandrosterone; sAA = salivary alpha-amylase; TPR = total peripheral resistance; CO = cardiac output

### Stress arousal reappraisal

Stress arousal reappraisal (SAR) makes use of the lay belief that stress is inherently negative. Experienced arousal (e.g., increased heart rate) during stressful situations (e.g., when giving a speech) is commonly perceived as debilitative and harmful towards task performance [39]. Consequently, a typical coping approach is to downregulate or eliminate stress arousal altogether [65]. Contrary to this misconception, SAR promotes the often-overlooked positive and adaptive aspects of stress responses and emphasizes an individuals’ agency in the emergence of said response. That is, the way people perceive stress (positive or negative) is in fact decisive to their stress responses [66]. Towards this end, SAR interventions instruct individuals to reinterpret stress arousal as a functional response, which is indeed beneficial for task performance. For instance, an increased heart rate prepares an individual for a demanding situation, by providing additional oxygen to the body and brain. The idea is that the act of defining stress arousal itself as a coping resource will cause a shift in the BPSM framework from threat towards challenge state, and ultimately promote more adaptive stress responses. It is noteworthy that task engagement is essential for reappraisal to thrive; if there is no sympathetic arousal to begin with, then there is nothing to reappraise [67]. In line with the suggested theory, previous research has demonstrated that SAR interventions lead to more efficient psychophysiological stress responses and improved task performance (e.g., [47, 48, 65, 68–71]).

### Worked examples

BBN is a particularly delicate and challenging situation, and therefore devoted training is of utmost importance for physicians to optimize their BBN-performance [72, 73]. The question of how to best teach communication skills remains a core issue for medical schools worldwide [74–76]. We postulate that worked examples (WE) are a suitable approach to teach established BBN communication protocols (e.g., SPIKES, [1]). A worked example usually implies an initial problem (in our case how to BBN to a patient) and provides a step-by-step demonstration of its successful solution (in our case showing a physician BBN by following six steps of SPIKES). WEs are most useful for novice learners with restricted prior knowledge [77]. By breaking down an ill-defined task into individual steps, the complexity of the task, and at the same time, the cognitive load of the learner can be reduced. This allows for an easier acquisition of schemas in long-term memory, which can then be utilized to approach similar problems (e.g., communication of various diagnoses) [78]. Schemas can be retrieved as a single unit from memory despite cognitive constraints that might also occur during BBN [79]. While WE interventions have proven to be effective in well-defined domains such as mathematics and physics [80, 81], more recently, similar effects were demonstrated in ill-defined domains such as developing a medical diagnosis [82–85]. By improving the acquisition of relevant BBN-related schemas, preparatory worked example-based learning should increase the perceived coping resources of medical students tasked with BBN to an SP and should therefore promote a shifting of their stress responses from threat to challenge.

### Hypotheses

The goal of this project is to determine how SAR and WE interventions influence (1) the psychophysiological stress response and (2) the communication-skills performance in medical students tasked with BBN in simulated settings. In addition, we will determine (3) if potential effects on communication performance are mediated by the psychophysiological stress response. Therefore, the study addresses following hypotheses:

H1.1 – Students receiving SAR instructions will exhibit significantly more adaptive, challenge-type psychophysiological responses (i.e., higher resources-demands differential, higher challenge-threat cardiovascular index, higher anabolic balance and higher sAA) than students receiving no SAR instructions.

H1.2 – Students receiving SAR instructions will show significantly better communication skills performance than students receiving no SAR instructions.

H2.1 – Students preparing themselves for the BBN task by learning from a BBN-related worked example will exhibit significantly more adaptive, challenge-type psychophysiological responses than students not preparatorily learning from a BBN-related worked example.

H2.2 – Students preparing themselves for the BBN task by learning from a BBN-related worked example will show significantly better communication skills performance than students not preparatorily learning from a BBN-related worked example.

H3.1 – The challenge-threat resources-demands differential and cardiovascular index are significant mediators of the effect of the SAR intervention on communication skills performance.

H3.2 – The challenge-threat resources-demands differential and cardiovascular index are significant mediators of the effect of preparatory worked example-based BBN learning on communication skills performance.

Furthermore, there are secondary, exploratory issues we want to address with this study. Theory suggests that SAR interventions might also improve mood [39]. However, research on this aspect is inconclusive [61] and the effects of WE on mood have not been investigated previously. Therefore, we treat the effects of SAR and WE on mood as an exploratory issue.

Stress mindsets are global belief systems about the nature of stress [86]. Individuals with a stress-is-enhancing mindset belief that stress is positive and promotes health and performance, whereas individuals with a stress-is-debilitating mindset belief that stress is negative and harms health and performance. By assessing stress mindsets before and after the interventions, we can check if the manipulations affected an individual’s perception of stress (e.g., [57]). This is especially interesting for medical students, since they already have a certain understanding of the functionality of stress responses presented in SAR. Pre-existing stress mindsets might be pivotal for the effectiveness of SAR interventions [67]. One previous study suggests that most cognitive benefits occur when an individual holds a stress-is-enhancing mindset and appraises a stressor as challenge [57]. However, one could also argue that instructing individuals with a stress-is-enhancing mindset to reappraise stress arousal as functional is redundant [67]. Therefore, we will (1) consider stress mindset as a control variable in the primary analysis and (2) analyze stress mindset as a secondary outcome, to evaluate if SAR interventions affect the stress mindset of medical students.

## Methods

### Participants

Participants will be German speaking third-year medical students from Swiss Universities, with no prior experience in BBN. They will be recruited through social media outlets, emails, and direct information in the lecture hall. Exclusion criteria are factors known to affect the psychophysiological outcomes: cardiovascular diseases, neuroendocrine conditions, use of psychotropic drugs or medication, and wearing a pacemaker. Female students additionally cannot participate if they are pregnant or lactating. Participants will be compensated with a monetary reward of 150 Swiss Francs for completing the study, and travel expenses will be reimbursed. Our goal is to collect valid data from 200 participants, to test the hypotheses with the necessary statistical power (see “Sample size calculation” for more details).

### Study design

**Fig. 1:**
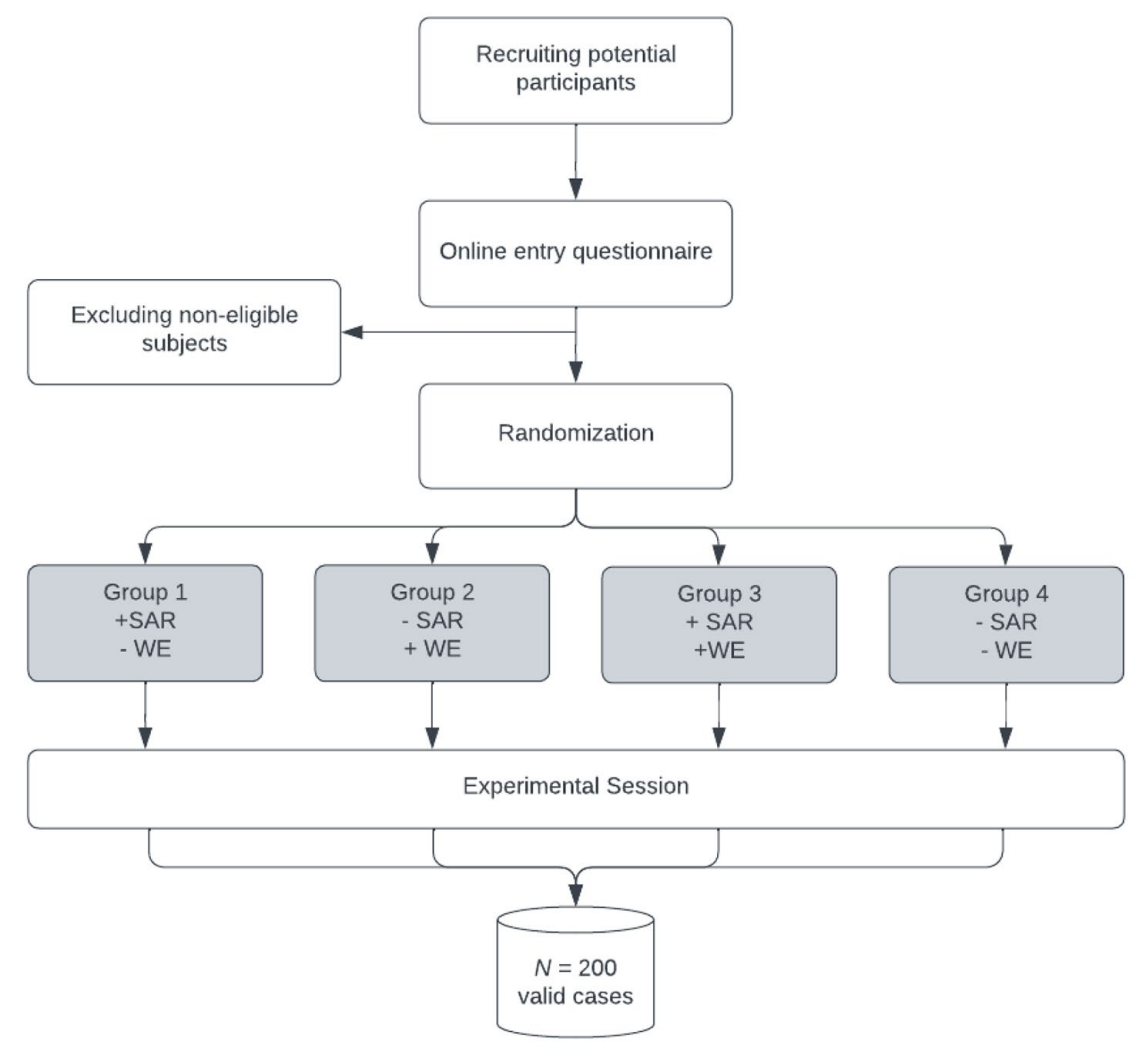
Flow of participants *Note*: SAR = Stress arousal reappraisal; WE = Worked example

To test our hypotheses, we will conduct a randomized controlled trial applying a 2 (SAR vs. control) x 2 (WE vs. control) between-subjects design. Participants will be stratified according to gender and randomly assigned to one of four conditions: (1) SAR only, (2) WE only, (3) SAR and WE, (4) no intervention (see Fig. 1). Participants will be blinded to their condition and will not be informed about the various interventions. Raters of the BBN-skills performance will receive video recordings of the BBN situations in a random order with no information about the assigned condition. SPs interacting with the participants will only be present for the BBN situation and will not be informed about which intervention was applied before the encounter. The experimenters will not be blinded, as they are required to set up the corresponding learning modules (including interventions) for each session.

### Study procedure

#### Online entry questionnaire

Interested students will be first invited to fill in an online entry questionnaire. The purpose of this questionnaire is to collect sociodemographic data as well as determine students’ eligibility. Eligible participants will then be scheduled for an individual experimental session in the research lab at the Institute for Medical Education in Bern.

#### Experimental session

The day before the experimental session, an email will be sent to the participants with the request to comply with the following instructions: no alcohol consumption and no intense physical activity 24 h before the session, no heavy meals and no caffeine consumption 2 h before the session, no smoking and no food consumption 1 h before the session.

**Fig. 2:**
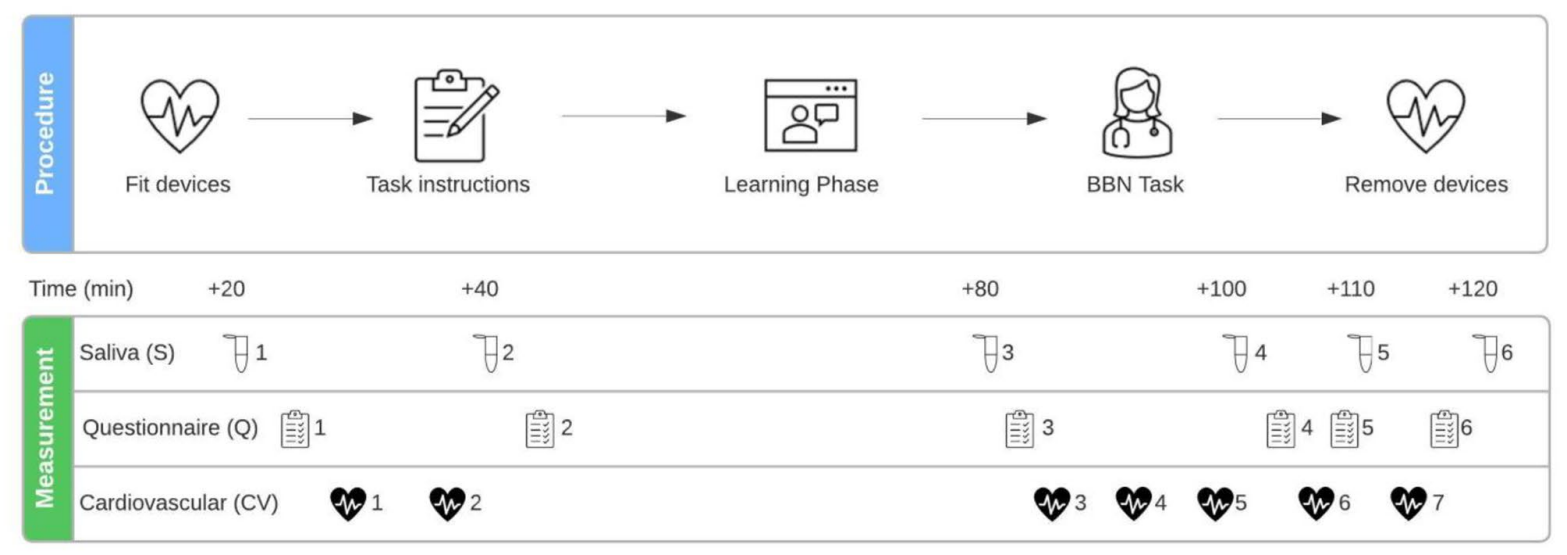
Experimental Session *Note*: Q1 contains the Multidimensional Mood State Questionnaire (MDMQ) and Stress Mindset Measure (SMM). Q2 contains the MDMQ, self-reported demands and resources, prior BBN experience and skills, and BBN interest and motivation. Q3 and Q4 both contain the MDMQ and self-reported demands and resources. Q5 includes the Depression, Anxiety and Stress Scale (DASS-21), Emotion Regulation Questionnaire (ERQ), SMM and MDMQ. Q6 contains the MDMQ

The 2-hours session will start at 2pm for all participants to control for the psychophysiological effects of the circadian rhythm. The timing of each step will be identical across conditions (see Fig. 2).

Upon arrival at the lab, the experimental session, the cardiovascular measurement instruments, and the saliva sampling procedure will be explained to the participants, and written consent will be obtained from them. When explaining the experimental session, the experimenter will specifically mention that the BBN task will be video recorded, based on which participants’ performance will be rated. This information aims to increase participants’ task engagement. After these explanations, participants will have to verify their compliance with the behavioral instructions (food intake, caffeine consumption, etc.). Any violation of the behavioral instructions will be noted and considered in the statistical analysis. Participants will then be fitted with the sensors of the cardiovascular measurement devices (Finometer and VU-AMS). Next, a first saliva sample (S1) will be collected and a questionnaire (Q1; see Fig. 2 for details) answered, followed by a cardiovascular baseline measurement of 5 min (CV1). After the baseline measurement, participants will be informed about the setting of the upcoming task (i.e., BBN in prenatal setting). This will be followed by a cardiovascular recording of 2 min (CV2), a second saliva sample (S2), and a second questionnaire (Q2). During the next 40 min, participants will learn how to BBN with a web-based tool that includes a short introduction to BBN and the SPIKES protocol [1]. This protocol divides BBN into six stages (i.e., Setting, Perception, Invitation, Knowledge, Emotion, Strategy & Summary). The assigned intervention (SAR and/or WE) or control material will be part of this learning module. Immediately after the learning period, another saliva sample will be collected (S3), and a third questionnaire will be answered (Q3). Participants will then receive detailed information about a diagnosis (Trisomy 21) and be given 5 more minutes to prepare, before they have to deliver the bad news to an SP. For the BBN task, participants will be given a time limit of 12 min but will be allowed to finish earlier. Cardiovascular data will be recorded for 2 min before (CV3), during (CV4), and for 2 min after the BBN task (CV5). Next, another saliva sample will be collected (S4), and a fourth questionnaire will be answered (Q4). For the remainder of the experiment, there will be two blocks consisting in cardiovascular recording (CV6, CV7), followed by questionnaires (Q5, Q6) and saliva samples (S5, S6). During the cardiovascular recordings (excluding the BBN task), participants will be asked to sit quietly in an upright position, to not cross their legs, keep their eyes open, and their hands on the table.

## Measures

### Online entry questionnaire measures

With the online entry questionnaire, the following sociodemographic control variables will be collected: age, gender, shift work (yes/no), and place of study. Additionally, to determine the eligibility of interested students, the following parameters will be gathered: current enrolment year, previous experiences in BBN, cardiovascular and neuroendocrine diseases, wearing a pacemaker (yes/no), use of medication and psychoactive drugs. Female participants will further provide information on the timing of their menstrual cycle and indicate whether they are pregnant, lactating, or use hormonal contraceptives.

### Experimental session measures

From the measures obtained during the experimental session, we will derive primary outcomes, which will be used for hypothesis testing, and secondary outcomes, which will be used as control variables in the primary analyses or for exploratory analyses.

#### Cardiovascular measures

We will use two different devices to measure cardiovascular activity.

The Finometer (FMS Finapres Medical Systems, Amsterdam, The Netherlands) measures finger arterial pressure on a beat-to-beat basis by finger-cuff photoplethysmography. The finger cuff is wrapped around the middle phalanx of the left middle finger. Hydrostatic height correction of the finger with respect to the heart level will be active during the measurement. The Finometer produces waveform measurements similar to intra-arterial recordings and reconstructs brachial arterial pressure. The resulting artery pressure wave will provide systolic blood pressure (SBP), diastolic blood pressure (DBP), and mean arterial pressure (MAP). Data recorded by the Finometer will be analyzed using Beatscope software. The Finometer will be paused during the learning phase, to relieve participants from prolonged pressure.

The VU Ambulatory Monitoring System (VU-AMS, Free University, Amsterdam, The Netherlands) consists of seven non-invasive electrodes, which will be applied on the participants’ thorax and back. The instrument is used to obtain impedance cardiographic and electrocardiographic data, namely, HR, PEP, left ventricular ejection time, stroke volume (SV) and CO. Data will be analyzed using the VU Data Analysis & Management Software (VU-DAMS).

The parameters derived from the two instruments are used to calculate the cardiovascular index of challenge and threat (z-scored CO-TPR), which represents a primary outcome used for hypothesis testing. CO is calculated by multiplying SV by HR (CO = SV*HR). TPR is then obtained by dividing MAP by CO (TPR = MAP/CO). In line with standard procedures in research using the BPSM of challenge and threat, HR and PEP will be analyzed to ensure that the sample as a whole was sufficiently engaged [25, 87].

A motion sensor embedded in the VU-AMS will record triaxial acceleration and angular velocity to quantify body motion, which will be used as a control variable.

#### Salivary measures

A passive drooling method will be used to collect saliva into low-bind polypropylene 2 mL cryovials (Salicap, IBL International, Hamburg, Germany). Before taking a sample, participants will be asked to swallow all saliva in their mouth, then accumulate saliva for 2 min, and finally transfer all saliva into the Salicap. After collection, the samples will be stored in a freezer at -30 °C. Free salivary cortisol (sC) and dehydroepiandrosterone (sDHEA) will be measured using a Cortisol and Dehydroepiandrosterone Saliva Luminescence Immunoassay kit (IBL-Tecan, Hamburg Germany), respectively. Salivary alpha amylase (sAA) activity will be measured using reagents provided by DiaSys Diagnostic Systems (Holzheim, Germany). Both the anabolic balance (sDHEA divided by sC) and sAA are primary outcomes used for hypothesis testing.

#### Self-reported demands and resources

Before the learning phase (Q2) and before the BBN task (Q3), we will assess perceived demands with the question “How demanding do you expect the BBN task to be?” and resources with the question “How able are you to cope with the demands of the BBN task?”. After the task (Q4), participants will report how they perceived the demands by answering the question “How demanding was the BBN task?” and their resources with the question “How able were you to cope with the demands of the BBN task?”. Participants will be asked to rate each item on a 6-point Likert scale, ranging from 1 “not at all” to 6 “extremely”. The items are adapted to the context of the BBN task from previous research [37, 88, 89]. The resources-demands differential (i.e., resource score minus demand score) represents a primary outcome used for hypothesis testing. Higher scores indicate more perceived resources compared to demands and therefore more challenge-oriented stress responses.

#### BBN-skills performance

Participants’ skills performances as shown during the BBN encounter will be scored by three independent raters based on video recordings. Raters will be trained on an adapted version of the SPIKES scale (see supplementary material) [90] and the ‘global Breaking Bad News Assessment Scale’ (glBAS) [91]. The adapted SPIKES scale contains one item for the steps Setting, Knowledge, Emotions, Strategy & Summary and two items for Perception and Invitation (for these two steps, the mean of the two items will be used). An additional item assesses non-verbal communication aspects. All items are rated on a bipolar five-point rating scale ranging from 1 to 5 (the higher the better). The glBAS contains 5 items [91]. Each item represents different domains of BBN and is rated on a 5-point Likert scale ranging from 1 “very good” to 5 “very poor”. We will reverse the values of the original glBAS items to match the SPIKES scale. For the SPIKES scale, the final score is the grand mean of all steps and the non-verbal communication item across the three raters. Each step as well as the non-verbal item will be weighted equally. For the glBAS, the final score will be the grand mean across the 5 items and three raters. Communication performance is a primary outcome used for hypothesis testing.

The raters’ training will be structured according to previous research [90] and given by FMS. During approximately three days of training, raters will be presented with an introduction to BBN and critical literature, and the assessment methods will be discussed. Raters will then independently score video examples covering low, mediocre, and high student BBN-related performances by using the two scales. Each evaluation run will be followed by a group discussion during which raters are requested to reach a consensus on evaluation results.

#### Mood

We will use the MDMQ short-scale [92, 93] to assess three dimensions of mood, following the conceptualization of Matthews et al. [94] and Schimmack et al. [95]: valence (bad/good), calmness (tense/calm), and energetic arousal (tired/awake). Valence and calmness are composed of 3 bipolar items each, whereas energetic arousal is measured by 2 items. The scale ranges from 1 to 8 with two opposing adjectives as anchors (e.g., “Right now I feel tired (1) / awake (8)”). The total score for valence and calmness ranges from 3 to 24, and for energetic arousal from 2 to 12. Half of the items are reverse scored. For the individual dimensions, higher scores represent more positive valence, higher calmness, and higher energetic arousal. Mood is a secondary outcome analyzed for exploratory purposes.

#### Stress mindset measure

To gauge the extent to which participants perceive stress as enhancing or debilitating, we will use a validated 4-item short version of the SMM [86]. We translated the items to German and conducted a back translation to control for quality (see supplementary material). Participants will rate each item on a 5-point scale ranging from 0 “strongly disagree” to 4 “strongly agree”. Half of the items are reversed, and the mean is calculated as index score. Higher scores correspond to a more stress-is-enhancing mindset. Stress mindset represents a control variable and secondary outcome used for exploratory analyses.

#### Depression, anxiety and stress

The DASS-21 [96, 97] measures the three emotional states depression, anxiety, and stress with 7 items each (total of 21 items). Participants indicate how each of the items applied to them over the past week. The scale ranges from 0 “Did not apply to me at all” to 3 “Applied to me very much or most of the time”. Scores are calculated for each of the three subscales. For each subscale, higher scores stand for a higher manifestation of the emotional state. The German version of the test possesses good psychometric properties [97]. Depression, anxiety, and stress will be treated as control variables.

#### Emotion regulation

We will assess participants’ habitual use of the two emotion regulation strategies expressive suppression (4 items; e.g., “I keep my emotions to myself”) and cognitive reappraisal (6 items; e.g., “When I want to feel more positive emotion (such as joy or amusement), I change what I’m thinking about”) in their daily life with the German version of the ERQ [98]. Each item is rated on a scale ranging from 1 “not at all” to 7 “completely”. The mean is calculated separately for each emotion regulation strategy. Participants’ habitual use of expressive suppression and cognitive reappraisal will be used as control variables.

#### Prior experience and skills in BBN

To control for possible differences in prior experience and skills in BBN, we developed a 3-item questionnaire (see supplementary material). Participants will have to state on 2 “yes/no” items if they have prior practical experience in or theoretical knowledge of BBN principles (if so, participants will need to specify in a free text field to what extend). On an additional item, participants will rate their perceived skill level in BBN from 1 “very low” to 7 “very high”.

#### BBN interest and motivation

Participants will report to what degree they are interested in BBN and motivated to do well at the BBN task using two 7-point scales (1 “very little interest” to 7 “very interested”; 1 “not at all motivated to perform well” to 7 “absolutely motivated to perform well”). Interest and motivation will be used as control variables.

### Interventions

**Fig. 3:**
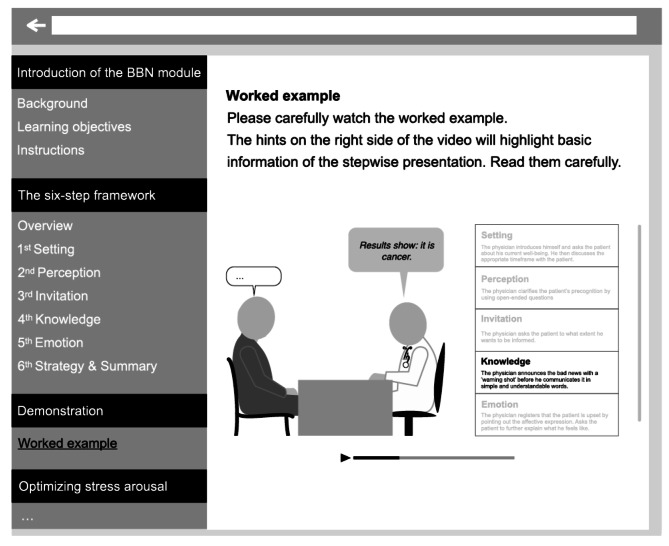
Illustration of the learning module *Note*: A general “Introduction of the BBN module” and “The six-step SPIKES framework” will be provided to participants of all groups. The worked example (“Demonstration”) and stress arousal reappraisal (“Optimizing stress arousal”) interventions will only be presented in the respective groups

The interventions will be part of the learning phase and complement a written version of the SPIKES protocol, which all participants receive, regardless of their condition (see Fig. 3).

### Stress arousal reappraisal

The content of the stress arousal reappraisal intervention is adapted from previous research [47, 61, 62, 69, 87, 99] and presented in the form of a 7-minute screencast using illustrations and voice-over. The screencast states that experiencing stress arousal is normal and legitimate and shows that an individual cares about a challenging situation. More specifically, participants will learn how stress arousal fulfills a vital function (e.g., an increased heart rate provides more oxygen to where it is needed) and is necessary for individuals to perform at their best. Therefore, stress arousal should be perceived as functional and beneficial for performance rather than harmful. To endorse the presented information, participants will then be asked to reflect on past and future stressful situations. Finally, right before the BBN communication task, participants will be reminded to reappraise the stress arousal they might experience in the encounter as beneficial for their BBN performance.

In the corresponding control condition, participants will be shown a 7-minute screencast about psychological and neurological learning processes (this is similar to control materials successfully used in previous studies, e.g., [87]).

### Worked example

The WE will be presented in the form of a 10-minute video showing a simulated BBN encounter between a physician and a patient. The BBN encounter will be structured according to the six-step SPIKES protocol, with the physician performing each step appropriately. For instance, in the step *Knowledge*, the physician first announces that they have bad news, before delivering the specific diagnosis (“Unfortunately, I do not have any good news for you today”). Each of the steps is accompanied by a written hint, emphasizing which step is being displayed and why it is done in this way (in accordance with Lorch [100]). During the same period of 10 min, participants attributed to the corresponding control condition will continue working with the written SPIKES protocol.

### Statistical analysis plan

To test the effectiveness of the interventions on communication performance (H1.2, H2.2), we will use 2 (SAR vs. no SAR) x 2 (WE vs. no WE) between-subjects ANOVAs. For the analysis of the repeated psychophysiological stress response outcomes (H1.1, H2.1), we will use linear mixed models with the participants as random effect. To test if the effects of the interventions on communication performance are mediated by the psychophysiological stress response (H3.1, H3.2), we will conduct a bootstrapping mediation analysis following state-of-the-art methodology [101]. We will follow international guidelines to derive the parameters used in the statistical analysis. For each cardiovascular measure, the mean of each 1-minute period will be calculated. For salivary measures, the area under the curve with respect to ground and increase will be computed. Where sensible, we will transform skewed variables to approach normality. We will perform sensitivity analyses with the control variables gender, age, BMI, use of hormonal contraceptives, shift work, SP, stress mindset, depression anxiety and stress, body motion, BBN-related interest & motivation, BBN-related prior experience & skills, and habitual use of emotion regulation strategies. For significance testing, an alpha level of 0.05 will be used.

### Sample size calculation

An a-priori power analysis was performed to calculate the required sample size, using the G*Power 3 program [102]. We reviewed published data on psychophysiological and performance effects of our two interventions, to determine that an effect size of *d* = 0.40 [103] is scientifically reasonable and practically relevant. Given an alpha level of 0.05 (two-tailed) and a targeted power level of 0.80, *n* = 50 participants per group will be required. Thus, we will need a total of *N* = 200 participants with valid data to test our hypotheses. In case of device malfunctioning, dropouts, non-compliance or unusable data, additional participants will be scheduled until a total of 200 valid cases is reached.

## Discussion

### Implications

We anticipate the findings of the current project to have several important implications for theory and practice. Findings will provide unique insights into the psychophysiology of medical students who are tasked with BBN. Psychophysiological stress responses elicited in BBN encounters might be meaningfully mapped on to and understood from the challenge and threat perspective. In a next step, differences in stress responses can be linked to the level of communication performance. Most importantly, we propose two short interventions—stress arousal reappraisal and worked examples—that might not only improve students stress responses, but also cause cascading effects on communication performance.

If proven effective, findings may open new avenues for innovations in medical education. Not only do we provide a novel and more comprehensive understanding of BBN encounters, but we also propose low threshold interventions that could be easily incorporated into the curriculum of medical students. Further, we expect the benefits of stress arousal reappraisal to spill over to similar stress-inducing communications with patients or other motivated performance situations, which are ubiquitous in education and the medical workplace environment (e.g., [48, 68, 104]). Finally, improved communication also benefits health related outcomes of concerned patients [14–20].

The current project might further contribute to the advancement of the BPSM of challenge and threat. For the first time, we will assess the well-established psychophysiological indices of challenge and threat (self-reported demands and resources, cardiovascular parameters), together with less often used indices of the activity of the HPA and SAM axes (sC, sDHEA, sAA), and performance outcomes. Therefore, we hope to provide a more comprehensive understanding of individuals’ psychophysiological and behavioural stress responses. To the best of our knowledge, this project is also the first to explore and evaluate challenge and threat parameters in the unique context of BBN encounters. By doing so, we expand the framework to motivated performance situations in the medical field.

### Possible pitfalls

We are aware that the present project is ambitious, which brings several possible challenges.

First, participation in the project will not be part of the regular medical study program but will rather be offered as an extracurricular learning opportunity. Although we assume that medical students have a general interest in communication training, we still rely on voluntary participation.

Second, medical students represent a distinct group of individuals compared to previous SAR-related research. In the third year, medical students already have a solid understanding of the functionality of their stress response, which is a core aspect of SAR interventions. Importantly, a person’s beliefs about stress (i.e., stress mindset) can affect their situation specific stress appraisal [67]. It remains to be seen how the SAR intervention applied in our study will affect the psychophysiology and performance of medical students specifically. We will investigate this issue by assessing participants’ stress mindset before and after the intervention and use it as control variable for primary analyses.

Third, there are more female medical students enrolled in Swiss universities than male students. Gender creates a possible confounder, given the different psychophysiology [87]. To ensure equal allocation to the experimental conditions, participants will therefore be stratified according to gender. Besides, the menstrual cycle of female participants causes monthly fluctuations of steroid hormones, which influence their psychophysiology. To control for these fluctuations, female participants will optimally be tested during the first week after menstruation. This puts restrictions on participation opportunities.

Lastly, while the BBN scenario is the same for all participants, for feasibility reasons, the SP role will be portrayed by different actors. To minimize any influence from SPs playing their role differently, a professional SP instructor of the Institute for Medical Education will provide detailed and identical training to each SP and ensure comparable acting. Further, SP will be considered as control variable in our statistical analyses. At the same time, because the BBN situation is only simulated, ecological validity could be questioned. Yet, due to the nature of the task, it is not feasible to conduct the experiment with real patients. It has been shown that communication skills performances exhibited in settings with SPs can predict performances in real clinical life [105].

## Electronic supplementary material

Below is the link to the electronic supplementary material.

Supplementary Material 1

## Data Availability

Not applicable.

## List of Abbreviations

BBN: Breaking bad news
BPSM: Biopsychosocial model
CO: Cardiac output
DASS-21: Depression Anxiety and Stress Scale
DHEA: Dehydroepiandrosterone
ERQ: Emotion Regulation Questionnaire
glBAS: Global Breaking Bad News Assessment Scale
HPA: Hypothalamic-pituitary-adrenal
HR: Heart rate
MAP: Mean arterial pressure
MDMQ: Multidimensional Mood State Questionnaire
PEP: Pre-ejection period
sAA: Salivary alpha-amylase
SAM: Sympathetic-adrenal-medullary
SAR: Stress arousal reappraisal
sC: Salivary cortisol
sDHEA: Salivary dehydroepiandrosterone
SMM: Stress Mindset Measure
SP: Simulated patient
SV: Stroke volume
TPR: Total peripheral resistance
WE: Worked example
